# First report of paternal uniparental disomy of chromosome 8 with *SLC52A2* mutation in Brown-vialetto-van laere syndrome type 2 and an analysis of genotype-phenotype correlations

**DOI:** 10.3389/fgene.2022.977914

**Published:** 2022-09-15

**Authors:** Siyu Zhao, Fengyu Che, Le Yang, Yanyan Zheng, Dong Wang, Ying Yang, Yan Wang

**Affiliations:** ^1^ Department of Pediatric neurology, Xi’an Children’s hospital, Xi’an, China; ^2^ Shaanxi Institute of Pediatric Diseases, Xi’an Children’s Hospital, Xi’an, China

**Keywords:** Brown-vialetto-van laere syndrome, SLC52A2, uniparental disomy, molecular diagnosis, genotype-phenotype correlations

## Abstract

**Purpose:** This study reports the clinical and genetic features of Brown-Vialetto-Van Laere syndrome (BVVL) type 2 in a case of uniparental disomy of chromosome 8 in mainland China and analyzes the genotype-phenotype correlation through a review of the literature of BVVL type 2 cases.

**Methods:** The clinical characteristics, treatment, and follow-up data of the patient were summarized, and the etiology was identified by whole-exome sequencing and gene chip analysis. Correlations between the genotype and phenotype were analyzed by collecting clinical and genetic data of published cases and our patient.

**Results:** We identified a homozygous mutation in *SLC52A2* (NM_001253815.2 c.1255G>A) by trio-WES. Sanger sequencing confirmed that his father was heterozygous and his mother was wild type. Subsequently, paternal uniparental disomy of chromosome 8 [UPD (8)pat] was confirmed by chromosomal microarray analysis.The patient received long-term oral riboflavin treatment (7 mg/kg.d) and was followed up for 40 months by which time the child’s bulbar palsy, ataxia, and motor function had improved. A review of the literature and statistical analysis found that the symptoms of BVVL type 2 appear at the earliest shortly after birth and at the latest at 10 years of age. The median age of onset was 2.5 years, but the overall delay in diagnosis was a median of 5.6 years. The most common symptoms were hearing loss (83.9%), followed by muscle weakness (80.6%), visual impairment (64.5%), and ataxia (61.3%). To date, a total of 32 mutations in the *SLC52A2* gene have been reported, with the most common being a missense mutation. Mutations occur throughout the length of the gene apart from at the N-terminus. In patients with missense mutations, homozygous pattern was more likely to present with ataxia as the first symptom (*p* < 0.05), while compound heterozygous pattern was more likely to develop respiratory insufficiency during the course of disease (*p* < 0.001). Moreover, patients with one missense mutation located in inside the transmembrane domain were more likely to have respiratory insufficiency than those with mutations both inside and outside the domain (*p* < 0.05). Riboflavin supplementation was an important factor in determining prognosis (*p* < 0.001).

**Conclusion:** We report the first UPD(8)pat with SLC52A2 homozygous pathogenic mutation case in BVVL type 2, which expand the mutation spectrum of gene.

## Introduction

Brown-Vialetto-Van Laere syndrome, also known as riboflavin transporter deficiency, is a rare neurodegenerative disorder. The disease was first described by Brown in 1894 (Brown, 1894)and subsequently by Vialetto (Vialetto, 1936)and Van Laere (Van Laere, 1966). BVVL has been renamed by riboflavin transporter deficiency (RTD) recently (Foley et al., 2014; Jaeger and Bosch, 2016b). The disease is clinically heterogeneous, and the age of onset can range from the neonatal period to adulthood. The clinical manifestations of the disease include sensorineural hearing loss, optic atrophy, bulbar palsy, gait ataxia, muscle weakness, axonal sensorimotor neuropathy, and respiratory disorders (O'Callaghan et al., 2019). To date, there are no epidemiological reports on the incidence of BVVL.

BVVL results from defects in riboflavin transporter function caused by mutations in *SLC52A2*(BVVL type 2) or *SLC52A3*(BVVL type 3). Riboflavin cannot be synthesized by body and must be obtained through intestinal absorption. Riboflavin is the precursor of the coenzymes flavin mononucleotide (FMN) and flavin adenine dinucleotide (FAD), which play important roles in biochemical redox reactions of carbohydrate, lipid, and amino acid metabolism (Yao et al., 2010; Haack et al., 2012; Ciccolella et al., 2013; Udhayabanu et al., 2016). As cofactors of several oxidoreductases, these enzymes play crucial roles in electron transfer in biological redox cycles and in maintaining the function of the normal respiratory chain (Yonezawa and Inui, 2013; Foley et al., 2014). In addition, they are involved in apoptosis and DNA repair (Spagnoli and De Sousa, 2012). The *SLC52A2* gene (NM_001253815.2), located at 8q24.3 and containing five exons, encodes a multi-pass transmembrane protein (RFVT2 protein) consisting of 445 amino acids with 11 transmembrane helices and is mainly expressed in the brain and spinal cord. The protein mediates cellular uptake of water-soluble vitamin B2/riboflavin and plays an important role in maintaining riboflavin homeostasis in the central nervous system (Yao et al., 2010). A total of 62 cases of BVVL type 2 have been reported throughout the world to date but there are no reports of uniparental disomy (UPD) or genotype-phenotype correlation studies. This study reports the first patient with BVVL type 2 due to UPD and uses published reports to analyze genotype-clinical phenotype correlations.

## Materials and methods

### The patient’s clinical features and laboratory results

**FIGURE 1:**
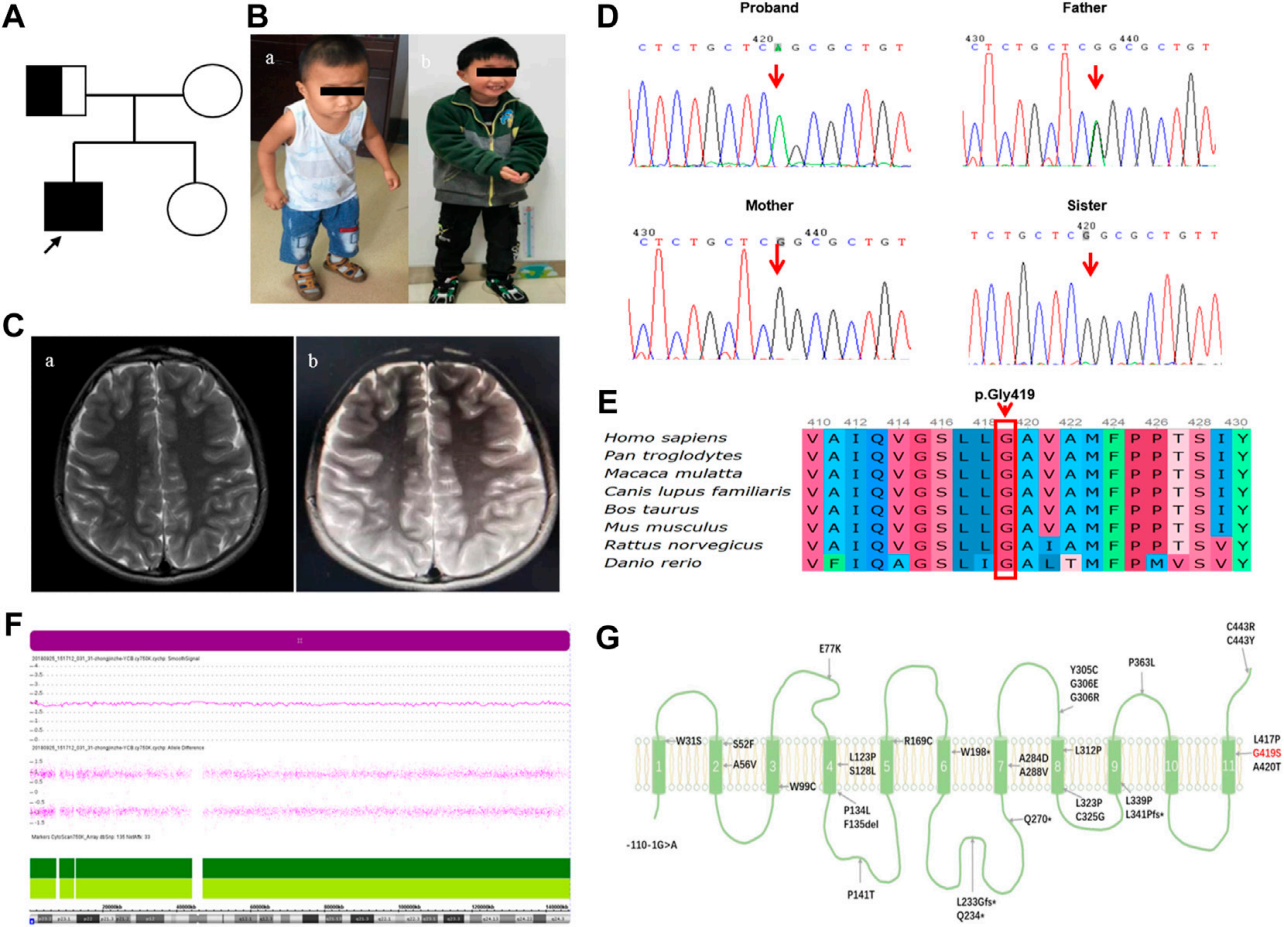
The patient’s clinical phenotype, neuroimaging features, and genetic outcomes are shown.**(A)** Pedigree map of BVVL caused by UPD(8)pat. **(B)** Clinical phenotype of a child with BVVL (Ba At the age of 3.4 years; Bb At the age of 6.8 years). **(C)** Brain MRI images of follow-up patients with BVVL (Ca showed normal head MRI at the age of 3.4 years; Cb showed bilateral parietal lobe T2WI high signal at the age of 6.8 years). **(D)** Sanger sequencing revealed a missense mutation in the *SLC52A2* gene in the proband (c. 1255G>A). His father was heterozygous, his mother was normal, and his sister was normal. **(E)** Conservation of the p.Gly419Ser variant founded in this study. **(F)** Array-CGH identified UPD (8) by showing LOH across chromosome 8. **(G)** Schematic diagram of RFVT2 protein transmembrane domain. The red font represents the mutation carried by the present case, while the black font represents the mutation reported in previous literature.

The proband was a boy, the first child of non-consanguineous parents of Chinese ancestry (Figure 1A). The patient was delivered at term with a normal birth weight [3,400 g (reference, 2500–4000 g)] and a normal head circumference [35 cm (reference, 31.8–36.3 cm)]. There were no significant issues in either the family or perinatal history. The patient’s early psychomotor development was normal. At 12 months, he could say redundancies such as “babamama”, walk on his own by 14 months, and run and jump by 24 months. At the age of 3.4 years, the child gradually developed visual acuity loss, and the visual acuity in both eyes was 0.025. Visual evoked potentials showed an absence of binocular pattern visual evoked potential (PVEP) although a binocular flash evoked visual potential (FVEP) was apparent. Although the patient had visual perception, the severe damage to the optic nerve limited the transmission of visual information to the visual center. At 42 months of age, the patient presented with hearing loss, which was followed by progressive muscle weakness, choking on drinking water, slurred speech, dysphagia, and inability to climb stairs and jump alone (Figure 1Ba). The patient had normal cognition and no seizures or sleep-disordered breathing. Neurological examination revealed nystagmus, dysarthria, hypotonia of the axial and limb muscles, decreased lower extremity reflexes, and gait ataxia with the feet set wide apart, a wider foot base, and small steps. Serum muscle and liver enzyme levels were normal, and serum ammonia (42.9 umol/L [reference, 9–30 umol/L])and lactate levels (2.43 mmol/L [reference, 0.7–2.1 mmol/L])were slightly elevated. MRI of the patient’s head and spinal cord was normal (Figure 1Ca). Blood count analysis revealed anemia (112 g/L [reference, 118–156 g/L]), decreased hematocrit and mean hematocrit, increased red blood cell volume, and increased mean hemoglobin concentration. Serum amino acid and acylcarnitine screening revealed mildly elevated levels of butyrylcarnitine [0.594 μm (reference, 0.060–0.500 μm)] and isovalerylcarnitine [0.347 μm (reference, 0.040–0.300 μm)]. Urine organic acid screening results were normal. Ear examination revealed severe sensorineural deafness in both ears. Electromyography showed axonal sensorimotor neuropathy with slowed motor nerve conduction velocity (MCV) and decreased compound muscle action potential amplitude (CMAP) in multiple peripheral nerves. After genetic examination, the patient was finally diagnosed with BVVL type 2 at age 4, and the patient was immediately treated with intravenous (iv) riboflavin 10 mg/kg.d. During the riboflavin infusion, the patient developed adverse gastrointestinal reactions such as loss of appetite, nausea, and vomiting, which he could not tolerate. Riboflavin was then given orally (10 mg/kg.d). The patient failed to return to the clinic regularly, and the final riboflavin level was maintained at 7 mg/kg.d for a long period. During this time, the patient’s sister, a healthy girl, was born. The patient was revisited at the age of 6.8 years after receiving continuous oral riboflavin, 7 mg/kg.d, for 40 months. At this time, the symptoms of bulbar palsy, such as drinking water, coughing, and dysphagia, had disappeared, the ataxic gait was significantly improved, the neck weakness and motor function were improved, and the patient was able to run and jump, although with slight clumsiness (Figure 1Bb). Hearing tests were performed while he was wearing hearing aids, and there was still severe sensorineural deafness in both ears. Repeat plasma metabolic screening showed that acylcarnitine levels had returned to normal. MRI of the head showed bilateral parietal T2 hyperintensity (Figure 1Cb). The patient was eventually treated with a cochlear implant because the hearing impairment did not improve.

### Genetic analysis

Three milliliters of whole blood were collected using EDTA as anticoagulant. A DNA extraction kit (Beijing, Tiangen) was used to extract genomic DNA. An Agilent capture kit, with the Illumina Cluster and SBS reagent were used for exon capture and enrichment. The enriched target gene fragments were sequenced using the Illumina HiSeq platform. Sequencing data were aligned to human reference genome sequences. Candidate mutations with a carrier rate of less than 0.5% were screened in the dbSNP, 1,000 Genomes, ESP6500, and ExAC databases. Candidate mutations potentially affecting protein coding or splicing were screened using the dbSNP, OMIM, HGMD, and ClinVar databases, and the conservation and potential pathogenicity of the candidate mutations were predicted using SIFT, PolyPhen2, and MutationTaster. Candidate variants were assessed for pathogenicity according to American Genetics and Genomics in Medicine (ACMG) guidelines. Finally, the candidate variants were verified by Sanger sequencing in the parents.

The patient’s mother gave birth to a second child in 2018, and Sanger sequencing was used to verify the candidate gene loci in this child at 16 weeks of gestation (amniotic fluid) and postpartum (blood).

### Chromosomal microarray analysis

An Affymetrix CytoScanTM 750K chip [containing 200,000 single nucleotide polymorphism (SNP) markers and 550,000 copy number variation (CNV) markers] was used. DNA from extracted from peripheral blood and was subjected to successive enzyme digestion, ligation, amplification, product identification, product purification, quantification, fragmentation, fragmentation quality control, labeling, hybridization, washing, staining, and scanning. The scan results were analyzed with Chas software and public databases such as Decipher, UCSC, OMIM, and DGV, as well as the internal database of our laboratory and related literature, were used to analyze CNVs and UPD.

### *SCL52A2* genotype-clinical phenotype correlation analysis

We used the HGMD database (www.hgmd.cf.ac.uk) to locate reports of BVVL type 2 patients with *SLC52A2* gene mutations. PubMed and Google Scholar searches were used to identify relevant articles with the keywords “BVVL”, “riboflavin transporter deficiency (RTD)”, “mutation”, “*SLC52A2*”, “hRFT3”, and “RFVT2”.Published cases of BVVL type 2 caused by mutations in the *SLC52A2* gene, together with the information on the present patient, were included in a literature review. The clinical characteristics, laboratory test results, imaging, gene mutation status, treatment and follow-up data of patients were collected, and the correlations between genotype and clinical phenotype were analyzed. We hypothesized that mutations resulting in defective protein products may have different phenotypic effects from mutations resulting in complete protein deletions (van Gassen et al., 2012). Mutations that may not produce a protein product or produce a severely truncated protein product were classified as class 1. This class includes nonsense, deletion insertion (out of frame), and splice-site mutations (except for mutations located within 50 bp of the last coding exon). Mutations that might still produce a protein product (albeit defective) were classified as class 2 (mainly missense and in-frame 3-nucleotide deletion mutations). First, the clinical phenotypic differences between class 1 and class 2 mutations of the *SLC52A2* gene were analyzed. Second, the missense mutations were classified using several different classification methods, and the differences in clinical phenotype and correlations between the different mutation classifications were analyzed. This was done as follows: 1) The missense mutations were divided into compound heterozygous variants and homozygous variants according to the alleles, and the differences in clinical phenotype between the two groups were analyzed; 2) The location of the transmembrane domain (TMD) in the sequence was predicted using TMHMM and ExPASy software, and the two missense mutations were divided into three groups based on their positions relative to the TMD. These groups were defined as: ① Both missense mutations were located within the TMD; ② One mutation was located within the TMD and the other outside the TMD, within either an intracellular or extracellular loop; ③ Both mutations were located outside the TMD. The clinical features associated with the three groups were analyzed; 3) TMHMM and ExPASy software were used to predict the NH2-terminal (N-terminal), COOH-terminal (C-terminal), and the middle region of *SLC52A2*. The middle region was defined as the part of the sequence that was neither at the C-terminal nor the N-terminal. The missense mutations were classified according to their position in the C-terminal, N-terminal, or middle regions of the sequence, and the clinical phenotypes were related to these positions.

### Statistical analysis

Enumeration data were described by frequency and percentage. Measurement data were expressed by median (Median, M) and interquartile range (Interquartile range, IQR). Statistical analysis was performed using the Mann-Whitney U test. The enumeration data were compared using the chi-square test. *p*-values less than 0.05 were considered statistically significant.

## Results

### Analysis of genetic variation results

The Trio-WES results showed that the child carried the *SLC52A2* gene NM_001253815.2 c.1255G>A (p.Gly419Ser) homozygous mutation; the father carried the heterozygous mutation, and the mother was wild-type at this locus (Figure 1D). Verification of the pedigree by Sanger sequencing was consistent with the Trio-WES results. This variant is not included in the dbSNP, ExAC, gnomAD, ESP6500, and 1,000 Genomes databases (PM2) and has been highly conserved during evolution (Figure 1E). SIFT, Polyphen2, and MutationTaster predicted that it may be detrimental (PP3), and *in vivo*/*in vitro* functional assays suggest that variants may lead to impaired gene function (PS3_supporting) (Ciccolella et al., 2013). This patient had a homozygous variant, and the pathogenic/suspected mutagenic variant c.155C>T has been reported at the trans position of this variant (PM3) (Ciccolella et al., 2013). Therefore, this variant was defined as “Likely Pathogenic” according to the ACMG guidelines (PM2+PP3+PS3_Supporting + PM3).

Further analysis of the whole-exome sequencing data of the family revealed that the patient had multiple homozygous variant loci on chromosome 8. Similarly, the father was heterozygous and the mother was wild-type. It is suggested that the homozygous variation may be due to paternal UPD on paternal chromosome 8. Subsequently, chromosome microarray analysis (CNV/aCGH) showed that the patient had a 146.124-kb loss-of-heterozygosity (LOH) region arr [hg19] 8p23.3q24.3 (168,483–146,292,734) over the entire chromosome 8 with the loci in the region being identical with the paternal loci, including the *SLC52A2* gene (Figure 1F). No copy number changes were found. Thus, combining the clinical phenotype and the molecular genetic findings, the proband was diagnosed with BVVL type 2, caused by a homozygous variant of *SLC52A2* with paternal UPD on chromosome 8.

### *SLC52A2* gene mutation spectrum

Supplementary Table and Figure 1G show the *SLC52A2* gene mutation information. Currently, a total of 62 BVVL type 2 patients from 43 different families were included, of which 40 had homozygous mutations and 22 had compound heterozygous mutations. A total of 32 mutations were found on 124 *SLC52A2* alleles (Table 1; Figure 1G). The most common type of mutation was a missense mutation (94.4%), followed by nonsense mutation (2.4%), splice-site mutation (0.8%), deletion mutation (1.6%, including 1 case of an in-frame deletion of 3 amino acids), and insertion mutation (0.8%). Pathogenic variants in *SLC52A2* were distributed throughout the gene in regions encoding the transmembrane domains and intracellular and extracellular loops. A total of 40.3% of the mutations were located in the transmembrane domain, 4.8% of the mutations were located intracellular loop, and 54.0% of the mutations were located extracellular loop. A total of 10.5% of patients had mutations in the C-terminal region, 89.5% were in the intermediate region, and no mutation was found at the N-terminal. Exon 3 had the most mutations (74.2%), followed by exon 5 (15.3%) and exon 4 (8.9%). The most common mutations were G306R (41.9%) and C443R (10.5%), accounting for almost one-half (52.4%) of all mutations with most detected in the homozygous state, followed by L339P (7.3%), R169C (4.8%), C325G (4.8%). The G306R mutation was most common in Lebanese, followed by Scots, possibly due to a founder effect.

**TABLE 1:** Comparison of the clinical characteristics of patients with *SLC52A2* gene homozygous mutations and compound heterozygous mutations.

	Homozygous (n = 40)	Compound heterozygous (n = 15)	*p* Value
Onset age (m) M(IQR)	36 (18–42)	24 (15.6–36)	0.634
Sex (M/F)	21/19	5/10	0.205
**Initial symptoms**			
Hearing loss	5 (12.5%)	5 (33.3%)	0.164
Visual loss	2 (5.0%)	2 (13.3%)	0.633
Nystagmus	6 (15.0%)	2 (13.3%)	1.000
Ataxia	24 (60.0%)	2 (13.3%)	0.002*
Weakness	0	2 (13.3%)	0.123
Pseudobulbar palsy	0	1 (6.7%)	0.273
Respiratory insufficiency	1 (2.5%)	1 (6.7%)	1.000
**Symptoms during course of the disease**			
Hearing loss	35 (87.5%)	14 (93.3%)	0.895
Visual loss	24 (60.0%)	14 (93.3%)	0.040*
Nystagmus	10 (25.0%)	3 (20.0%)	0.974
Weakness	29 (72.5%)	15 (100.0%)	0.058
Ataxia	31 (77.5%)	4 (26.7%)	0.0005*
Pseudobulbar palsy	15 (37.5%)	7 (46.7%)	0.537
Respiratory insufficiency	7 (17.5%)	13 (86.7%)	0.000002*

Statistically significant P values were marked in bold.

### *SLC52A2* clinical phenotype profile

Supplementary Table S1 summarizes the clinical characteristics and related pathological results of patients with BVVL type 2. The proportion of male and female patients was roughly the same. The disease begins at birth at the earliest and the latest at the age of 10. The median age of onset was 2.5 years, and the vast majority of patients had delayed diagnosis. The median age at diagnosis was 9 years, and the latest diagnosis was 54 years. Delay in diagnosis ranged from weeks to years, with an overall diagnosis delay of 5.6 years.

Among the 61 patients with BVVL type 2 with complete data on initial symptoms, the most common initial symptom was ataxia (44.3%), similar to that reported by Foley et al. (Foley et al., 2014). This was followed by hearing loss in 11 cases (18.0%), nystagmus in 9 cases (14.8%), muscle weakness in 4 cases (6.6%), and vision loss in 4 cases (6.6%). Nystagmus is often a warning sign of ocular disease in BVVL type 2, and patients with this sign are often found to have reduced vision and optic atrophy at the time of presentation (Foley et al., 2014; Gahl et al., 2016). In the course of the disease, 83.9% of patients developed hearing loss, manifested as bilateral, sensorineural damage. A total of 80.6% of patients developed muscle weakness, of which 56% had an axial weakness. Axial muscle weakness is often manifested as head drooping with forward-leaning of the upper body in the early phase. Muscle weakness of the upper limbs is often greater than that in the lower limbs during the early stage. As the disease progresses, the weakness in the lower extremities and the axial muscle weakness worsen, eventually leading to an inability to walk. A total of 64.5% of patients had visual impairment, with some showing optic atrophy (59.7%) and nystagmus (21.0%). In all, 61.3% of the patients had ataxia, often secondary to sensory neuropathy while 37.1% of patients developed respiratory insufficiency, of which 30.4% required mechanical ventilation. Other abnormalities were bulbar dysfunction in 40.3% of patients, intellectual disability in 11.3%, epilepsy in 3.2%, and breath-holding spells in 4.8% of patients.

Thirteen patients had complete blood analysis of amino acid metabolism, of which 3 patients (23.1%) had mild non-specific elevations. Of the 31 patients with complete blood carnitine testing data, 21 (67.7%) were abnormal.These 21 patients showed mild elevation of multiple acylcarnitines, with over two-thirds of them having a similar blood metabolic profile to multiple acyl-CoA dehydrogenase deficiency (MADD). Blood carnitine levels returned to normal after riboflavin supplementation. A total of 16 cases underwent urinary organic acid metabolism screening, of which 7 cases (43.8%) were abnormal and slightly elevated. Among them, 5 cases (71.4%) were found to have elevated urinary levels of ethylmalonic acid (ethylmalonic acid), similar to the urinary organic acid metabolism profile of short-chain acyl-CoA dehydrogenase deficiency (SCAD).

Of the 32 patients with complete head MRI results, the vast majority were normal, and only 6 (18.8%) were abnormal. Structural abnormalities (83.3%) were more common than signal abnormalities (16.7%). The structural abnormalities seen on brain MRI included defects in the corpus callosum, reduced vault volumes, and cerebellar atrophy, especially in the vermis of the cerebellum, while the observed signal abnormalities were signal enhancements in cranial nerves III and V. Spinal cord MRI data were present in four cases, two of which were abnormal and all of which showed signal changes. The patients presented with segmental or total spinal cord T2 signal increase with thickening and enhancement of the cauda equina root. Among the follow-up BVVL type 2 patients, only one patient underwent re-examination of head imaging (the present case), which was mainly manifested as bilateral parietal T2 hyperintensity, suggesting demyelination.

A total of 44 patients underwent electromyography, all of which were abnormal. Among the 42 patients with complete EMG data, 7 (16.7%) had pure sensory nerve damage, 10 (23.8%) had motor nerve damage, and 25 (59.5%) had mixed sensory-motor damage. Axonal injury was clearly demonstrated in 33 patients, while axonal injury data are unavailable in the remaining patients. Repeated EMG testing evidence of sensory neuropathy clearly preceded motor neuropathy, with all patients showing the same distribution pattern of motor neuropathy, with more severe involvement of the upper extremities than the lower extremities (Foley et al., 2014). A total of 13 patients underwent neuromuscular biopsies. Of these, eight patients with a complete gastrocnemius nerve biopsy showed the neuropathological manifestations of chronic axonal neuropathy with predominantly myelinated nerve fibers, and no signs of inflammation, pathological hypomyelination, or demyelination. In addition, among the six patients with complete gastrocnemius biopsy, the muscle pathological manifestations were non-specific, with most patients showing neurogenic muscle atrophy and disproportionate thickness of the muscle fibers, possibly accompanied by lipid accumulation.

### Correlation between clinical phenotype and genotype

A total of six cases of *SLC52A2* gene class 1 mutations were compound heterozygous mutations. Among them, one patient carried both a deletion and a missense mutation (note: P23 patient was not included), one patient had a splice site variant and a missense mutation, three patients carried a nonsense and a missense mutation, and one patient carried insertional and missense mutations. There were 56 patients in the *SLC52A2* gene class 2 group (mainly missense mutations and one case of a 3-nucleotide in-frame deletion mutation). Due to the small number of patients with class 1 mutations in the *SLC52A2* gene, no further genotype-phenotype correlations could be distinguished in this group of patients. However, among patients with class 1 mutations in *SLC52A2*, those with splice site variants appeared to have an earlier onset of symptoms. Karakaya et al. reported that a patient with a compound heterozygous *SLC52A2* gene (splice site variant and missense mutation) had postnatal onset with extensive muscle weakness and respiratory dysfunction requiring ventilator-assisted breathing maintenance (Karakaya et al., 2018).

Genotype-phenotype correlation analysis was performed on patients with double missense mutations in *SLC52A2*. First, we compared homozygous and compound heterozygous mutations. There were no significant differences in age of onset and sex between the two groups. In the patients with homozygous mutations, ataxia was the most common initial symptom and more common in the course of the disease (60.0 vs. 13.3% *p* < 0.05; 77.5 vs. 26.7% *p* < 0.001). Compound heterozygous patients more commonly showed visual impairment (93.3 vs. 60.0%, *p* < 0.05) and respiratory insufficiency (86.7 vs. 17.5%, *p* < 0.001, Table 1).

Second, we analyzed the relevance of mutation location in the transmembrane domain to the clinical phenotype. We found that there were no significant differences in the age of onset and sex among the three groups of patients. Compared with patients with all mutations in extra-TMD regions, hearing loss was more common initial symptom in patients with either all their mutations in the TMD or one mutation in the TMD (31.3 vs. 3.3%, *p* < 0.05; 44.4 vs. 3.3%, *p* < 0.05). Ataxia was the most common first symptom in patients having two extra-TMD mutations (70.0 vs. 25.0% vs. 11.1%, *p* < 0.05). Visual impairment and respiratory insufficiency occurred in different frequencies in the three groups. The incidence of visual impairment in patients with intra-TMD mutations was significantly higher than in those with extra-TMD mutations (87.5 vs. 53.3%, *p* < 0.05). The frequency of respiratory insufficiency was significantly higher in patients with one mutation within the TMD than in patients with both mutations either located within or outside the TMD. (88.9 vs. 31.3%, *p* < 0.05; 88.9 vs. 23.3%, *p* < 0.05). Patients with both mutations located in extra-TMD regions were more likely to develop ataxia symptoms over the course of the disease than those with only one mutation in the TMD (76.7 vs. 22.2%, *p* < 0.05). There were no significant differences in the frequencies of hearing impairment, nystagmus, muscle weakness, and bulbar disorders among the three groups of patients (Table 2).

**TABLE 2:** Comparison of the clinical characteristics of patients with missense mutations in the different transmembrane regions of the *SLC52A2* gene.

	Both mutations within TMD group 1 (n = 16)	One mutation within TMD group 2 (n = 9)	Both mutations outside TMD group 3 (n = 30)	Total *p* values	*p* Value G1:G3	*p* Value G1:G2	*p* Value G2:G3
Onset age (m) M(IQR)	24 (12–96)	36 (24–54)	30 (18–36)	0.727	0.837	0.519	0.475
Sex (M/F)	6/10	2/7	18/12	0.089	0.146	0.734	0.108
**Initial symptoms**							
Hearing loss	5 (31.3%)	4 (44.4%)	1 (3.3%)	0.005*	0.027*	0.821	0.008*
Visual loss	2 (12.5%)	1 (11.1%)	1 (3.3%)	0.464	0.567	1.000	1.000
Nystagmus	2 (12.5%)	0	6 (20.0%)	0.316	0.817	0.520	0.351
Ataxia	4 (25.0%)	1 (11.1%)	21 (70.0%)	0.001*	0.004*	0.755	0.006*
Weakness	0	2 (22.2%)	0	0.025*	—	0.120	0.049*
Pseudobulbar palsy	1 (6.3%)	0	0	0.455	0.348	1.000	—
Respiratory insufficiency	0	1 (11.1%)	1 (3.3%)	0.381	1.000	0.360	0.413
**Symptoms during course of the disease**							
Hearing loss	13 (81.3%)	8 (88.9%)	28 (93.3%)	0.359	0.449	1.000	0.556
Visual loss	14 (87.5%)	8 (88.9%)	16 (53.3%)	0.016*	0.020*	1.000	0.125
Nystagmus	6 (37.5%)	0	7 (23.3%)	0.101	0.501	0.105	0.269
Weakness	11 (68.8%)	9 (100.0%)	24 (80.0%)	0.172	0.625	0.176	0.351
Ataxia	10 (62.5%)	2 (22.2%)	23 (76.7%)	0.012*	0.501	0.129	0.010*
Pseudobulbar palsy	7 (43.8%)	3 (33.3%)	12 (40.0%)	0.878	0.806	0.932	1.000
Respiratory insufficiency	5 (31.3%)	8 (88.9%)	7 (23.3%)	0.001*	0.818	0.019*	0.002*

Statistically significant P values were marked in bold.

In addition, among 55 patients with double missense mutations in *SLC52A2*, six patients (10.9%) had mutations in the C-terminal region, all of which were homozygous C443R mutations, while 49 patients (89.1%) had mutations located in the intermediate region, and no mutations were seen in the N-terminal region. Patients with missense mutations at the C-terminus had an earlier age of onset (*p* < 0.05) and were more likely to have nystagmus as the first symptom (83.3 vs. 6.1%, *p* < 0.001). In these patients, nystagmus, decreased hearing and visual acuity, and muscle weakness occurred frequently, with none of the patients showing respiratory insufficiency (Table 3).

**TABLE 3:** Comparison of the clinical characteristics of patients with missense mutations in different positions in the *SLC52A2* gene.

	C-terminal (n = 6)	Middle region (n = 49)	*p* Value
Onset age (m) M(IQR)	15 (4–25)	36 (18–43.5)	0.006*
Sex (M/F)	3/3	23/26	1.000
**Initial symptoms**			
Hearing loss	0	10 (20.4%)	0.508
Visual loss	1 (16.7%)	3 (6.1%)	0.379
Nystagmus	5 (83.3%)	3 (6.1%)	0.000092*
Ataxia	0	26 (53.1%)	0.043*
Weakness	0	2 (4.1%)	1.000
Pseudobulbar palsy	0	1 (2.0%)	1.000
Respiratory insufficiency	0	2 (4.1%)	1.000
**Symptoms during course of the disease**			
Hearing loss	6 (100.0%)	43 (87.8%)	1.000
Visual loss	5 (83.3%)	33 (67.3%)	0.740
Nystagmus	5 (83.3%)	7 (14.3%)	0.001*
Weakness	2 (33.3%)	33 (67.3%)	0.236
Ataxia	6 (100.0%)	38 (77.6%)	0.449
Pseudobulbar palsy	3 (50.0%)	19 (38.8%)	0.930
Respiratory insufficiency	0	20 (40.8%)	0.130

Statistically significant P values were marked in bold.

The most frequently reported mutation in BVVL type 2 patients is c.916G>A (p.G306R), which was present in 28 patients. Homozygous mutations were the most frequently reported (71.4%), mostly in patients of Lebanese ancestry. A total of 8 cases (28.6%) were compound heterozygous. There were no significant differences in age of onset and sex between the two groups. Ataxia was the most common initial symptom and more common in the course of the disease in patients with the G306R homozygous mutation (95 vs. 12.5%, *p* < 0.001; 95 vs. 12.5%, *p* < 0.001). During the disease, compound heterozygous patients had a higher frequency of respiratory insufficiency than those with homozygous mutations (87.5 vs. 25.0%, *p* < 0.001). The G306R homozygous mutation shows clinical heterogeneity, with the earliest age of onset at 18 months (infancy) and the latest in childhood (96 months). During the disease, audio-visual disturbances, muscle weakness, and ataxia symptoms were common, and some patients may have bulbar disturbances and respiratory insufficiency (Table 4).

**TABLE 4:** Comparison of the clinical features between the homozygous G306R mutation and compound heterozygous mutations in the *SLC52A2* gene.

G306R	Homozygous (n = 20)	Compound heterozygous (n = 8)	*p* Value
Onset age (m) M(IQR)	36 (30–42)	24 (19.5–45)	0.418
Sex (M/F)	11/9	3/5	0.676
**Initial symptoms**			
Hearing loss	1 (5.0%)	2 (25.0%)	0.188
Visual loss	0	1 (12.5%)	0.286
Nystagmus	0	1 (12.5%)	0.286
Ataxia	19 (95.0%)	1 (12.5%)	0.000052*
Weakness	0	2 (25.0%)	0.074
Pseudobulbar palsy	0	0	—
Respiratory insufficiency	0	1 (12.5%)	0.286
**Symptoms during course of the disease**			
Hearing loss	18 (90.0%)	7 (87.5%)	1.000
Visual loss	10 (50.0%)	7 (87.5%)	0.099
Nystagmus	1 (5.0%)	1 (12.5%)	0.497
Weakness	19 (95.0%)	1 (12.5%)	0.000052*
Ataxia	14 (70.0%)	8 (100.0%)	0.216
Pseudobulbar palsy	7 (35.0%)	3 (37.5%)	1.000
Respiratory insufficiency	5 (25.0%)	7 (87.5%)	0.009*

Statistically significant P values were marked in bold.

### Riboflavin treatment and efficacy

Before treatment, the patients showed varying levels of decline or loss of motor function. Two (3.2%) patients never gained the ability to walk during the motor developmental milestones, 14 (22.6%) required assistance to walk, and 13 (21.0%) lost the ability to walk. The median age for loss of walking ability was 5 years (minimum 1.25 years, maximum 25 years). A total of 38 patients with BVVL type 2 received riboflavin. The median age for initiating riboflavin treatment was 9 years (the minimum was 1.7 years, the maximum was 52 years), and the therapeutic doses ranged from 7–70 mg/kg.d. No serious adverse reactions were observed during the treatment period, and treatment was only discontinued in a small number of patients due to adverse gastrointestinal reactions. Among the 53 patients with complete follow-up data, the follow-up periods ranged from 2 weeks to 6 years (median 7 months). No patients died during the observation period of riboflavin treatment (*p* < 0.05, OR 4.18 95%CI: 2.50–7.00, Table 5). The disease course was delayed in most patients treated with riboflavin. A total of 21 patients (55.3%) showed improvement in symptoms, mostly seen in increased muscle strength and improved gait in ataxia. In addition, some patients’ bulbar function, hearing, and vision improved. The remaining patients showed stable symptoms after riboflavin supplementation. A total of nine (14.5%) patients died, none of whom had been treated with riboflavin. The median age of death was four years (the youngest was 1 year, the oldest was 22 years). The causes of death included respiratory failure in two cases, gastroenteritis in one case, and the remaining six cases were not described in detail.

**TABLE 5:** Association of clinical features with prognosis.

	Death (n = 9)	Survival (n = 46)	*p* Value
Onset age (m) M(IQR)	30 (11–30)	36 (18–42)	0.265
Sex (M/F)	6/2	21/24	0.274
**Symptoms during course of the disease**			
Hearing loss	6 (66.7%)	41 (89.1%)	0.218
Visual loss	4 (44.4%)	32 (70.0%)	0.286
Nystagmus	3 (33.3%)	9 (19.6%)	0.636
Weakness	5 (55.5%)	28 (60.9%)	1.000
Ataxia	7 (77.8%)	38 (82.6%)	1.000
Pseudobulbar palsy	5 (55.5%)	18 (39.1%)	0.586
Respiratory insufficiency	4 (44.4%)	16 (34.8%)	1.000
**Mutation type** homozygous	7 (77.8%)	30 (65.2%)	0.729
**Riboflavin supplementation** (yes/no)	0/9	35/11	0.000075*

Statistically significant P values were marked in bold.

## Discussion

This study is the first to report a patient with BVVL type 2 caused by a paternal uniparental diploid *SLC52A2* mutation. The patient’s *SLC52A2* gene showed a homozygous mutation of c.1255G>A (p.Gly419Ser). The patient first developed vision loss at the age of 3.4 years. The subsequent disease progression was gradual, with the appearance of bilateral sensorineural hearing impairment, muscle weakness, ataxia, and bulbar disturbances. Ciccolella et al. previously reported a case of a rapidly progressive lethal case caused by a compound heterozygous mutation in the *SLC52A2* gene in a patient with the c.1255G>A mutation (another variant was c.155C>T). The patient’s disease progressed rapidly, beginning at 2 years of age in infancy. The patient’s symptoms included progressive dysphonia, marked exercise intolerance, dyspnea, and cyanosis. The patient developed vision and hearing loss, limb weakness, and walking disorders at the age of 3 years and died after admission due to respiratory disorder and aspiration pneumonia. Investigation of patient fibroblasts showed significant reductions in the expression and distribution of the RFVT2 protein, with riboflavin uptake and transport in only 29% of those in the control group. However, the manner in which the mutation affected riboflavin transport and membrane expression was not studied (Ciccolella et al., 2013). Our patient had a homozygous mutation c.1255 G>A in the *SLC52A2* gene, which was located in the 11th transmembrane domain of the RFVT2 protein. This mutation at residue 419 results in a change from the non-polar hydrophobic glycine to the polar neutral serine, which may lead to changes in protein conformation, potentially disrupting protein-protein interactions and transporter-membrane interactions, as well as adversely affecting the passage of substrate through the membrane, leading to reduced riboflavin transport and uptake capacity.

The UPD can cause disease not only through imprinting defects but also through homozygous exposure to recessive genes (Chen et al., 2021). It is currently believed that imprinted genes exist on chromosomes 6, 7, 11, 14, 15, and 20 (Patten et al., 2014). There have been several previous reports of UPD on chromosome 8 (Matsubara et al., 2014; Safka et al., 2017; Tachibana et al., 2022; Yu et al., 2022). For example, Tachibana N, et al. reported a patient with retinitis pigmentosa due to maternal whole UPD (8) with homozygous mutation of *PR1* gene. UPD was suspected because the patient’s mother carried the mutation and multiple SNPs on the chromosome were the same as the patient’s, which was finally confirmed by Microarrays (Tachibana et al., 2022). Matsubara K et al. also reported a patient with congenital adrenal hyperplasia due to paternal whole UPD (8) with homozygous mutation of *CYP11B1* gene (Matsubara et al., 2014). The above two patients showed only the clinical phenotype of primary disease, without other additional phenotypes, as did our patient. These findings support the hypothesis that UPD (8) does not cause specific imprinting disorders, and its disease phenotype often results from the homozygous exposure of recessive genes. Trio-WES has been widely used for identifying the etiology of rare diseases. If the patient carries a homozygous mutation with loss of heterozygosity (LOH) in SNPs in the chromosomal region near the mutation site, and one parent is a carrier, UPD should be considered. This can be confirmed by Microarrays. Since UPD is essentially a rare occasional mitotic error, its prevalence in siblings can be considered negligible (Ding et al., 2012). This suggests that genetic counseling is not necessary, confirmed by the fact that the sister of the present patient was healthy.

In this paper, we have also summarized the data of 62 patients from 43 families around the world diagnosed with mutations in the *SLC52A2* gene, to determine the correlation between clinical phenotype and genotype. This showed that the disease may present at all ages, with the age of onset ranging from birth to 54 years. BVVL type 2 caused by mutations in *SCL52A2* form a continuum extending from severe, fatal cases in infants (Naess et al., 2021) to some of the least affected cases with only ataxia symptoms (Fogel et al., 2014). The disease tends to progress gradually but can also be rapidly progressive. The most common initial presenting symptom of BVVL type 2 is often ataxia, similar to the findings of Foley (Fogel et al., 2014), followed by hearing impairment. Hearing impairment is often insidious, and the slight decline in the early stages is often ignored by parents, and moderate or above bilateral sensorineural hearing impairment is often present at the time of consultation. Nystagmus is often an early warning sign of ocular disease in BVVL type 2. Nystagmus is often accompanied by decreased vision and optic atrophy. As the disease progresses, most patients progressively display a variety of symptoms, including hearing impairment, muscle weakness, visual impairment, ataxia, and bulbar dysfunction. A lack of awareness of the disease often leads to delays in diagnosis. In the early stage of the disease, patients are often misdiagnosed with mitochondrial disease, spinal muscular atrophy, hereditary spinal ataxia, and other diseases. Diagnosis of BVVL type 2 was observed to be delayed by an average of more than 5.6 years, with the latest only being diagnosed at the age of 54. Therefore, when patients have more than two of the above symptoms, clinicians should be alert to the possibility of BVVL type 2.

Riboflavin deficiency may lead to a deficiency of many enzymes involved in amino acid and fatty acid metabolism and respiratory chain function, resulting in a relatively slight increase in metabolites associated with these enzymes (Woodcock et al., 2018). Animal experiments have found that severe riboflavin deficiency in rats mimics the metabolic signature of polyacyl-CoA dehydrogenase deficiency (MADD) (Goodman, 1981; Haack et al., 2012). Nearly 66.7% of patients had mild elevations in blood acylcarnitines, and these elevations were nonspecific. The abnormal acylcarnitine profile was observed to normalize after riboflavin supplementation, thus serving as a suggestive clue to riboflavin transporter deficiency. The vast majority of patients with *SLC52A2* mutations have normal brain MRI, whole patients with abnormal MRI may show structural abnormalities, enhanced cranial nerve signals, and signal changes in the spinal cord. In this case, the early brain MRI was normal, while the brain MRI T2 showed a high signal during the re-examination, suggesting the presence of demyelination. EMG indicated that axonal injury and demyelination were present in the peripheral nerves of our patient. A review of the literature revealed the identification of axonal neuropathy on sural nerve biopsies of patients with BVVL type 2. The neuropathy preferentially affected large-caliber myelinated axons, and muscle biopsies often showed denervation (Foley et al., 2014; Srour et al., 2014; O'Callaghan et al., 2019). This is consistent with the EMG findings suggesting that the axonal damage mainly involved peripheral nerves. Riboflavin is an important cofactor for myelin synthesis. Previous animal studies have shown that severe riboflavin deficiency may lead to the destruction of the myelin lamina, nerve fiber plasma membrane, axonal membrane, and progressive pathological myelin degeneration or demyelination. Riboflavin deficiency can also lead to mitochondrial dysfunction and ultrastructural abnormalities (Colasuonno et al., 2020). Model mice with mitochondrial dysfunction may exhibit primary Schwann cell and myelin dysfunction leading to axonal degeneration (Viader et al., 2013). The mechanism of axonal injury caused by riboflavin deficiency needs to be further studied.

To date, 32 different *SLC52A2* mutations have been identified, and multiple mutation types can occur. The most common are missense mutations, which are located in regions other than the N-terminus. Many nutrient/substrate transporters contain sequences/regions important for their function and targeting to cell membranes at their N-terminus (Suzuki et al., 2001; Subramanian et al., 2010). Subramanian VS. et al. found that the N-terminus of RFVF2 plays an important role in plasma membrane targeting and distribution. Complete deletion of the C-terminal region of RFVT2 resulted only in impairments to riboflavin transport whereas deletion of the RFVT2 N-terminal sequence, adversely affecting membrane targeting accompanied by a significant decrease in RFVT2 protein stability/translational efficiency and riboflavin uptake (Subramanian et al., 2015). The RFVT2 protein is essential for the survival of embryonic cells *in vivo*, and the article have reported that *SLC52A2* knockout is lethal at the embryonic stage in animal models (Jin et al., 2021). Thus, it is speculated that mutations in the *SLC52A2* N-terminus may be lethal due to the severity of the phenotype. Compared with missense mutations, splice-site mutations appear to have earlier onset and result in more severe disease. However, due to the small number of cases of this type and the incomplete data of some patients, the correlation between the two cannot be determined. *SLC52A2* missense mutations located in the C-terminal regions tend to result in an early age of onset, with nystagmus as the first symptom. To date, no cases of respiratory insufficiency have been reported among these patients. A combination of missense mutations in a compound heterozygous pattern, or within or outside of the transmembrane domain is more likely to lead to respiratory insufficiency. For these patients, the synergistic effect (or inheritance) of the variants on two riboflavin transporter genes could explain the phenotype, and it was also demonstrated that the free combination of the two mutant loci further complicates the clinical phenotype of the patients. G306R is the most common mutation type in BVVL type 2, and the homozygous mutation is mostly seen in patients of Lebanese ancestry. Patients with the G306R homozygous mutation have a high incidence of ataxia, and patients with G306R compound heterozygous mutations have a high incidence of respiratory insufficiency. In addition, there are differences in clinical phenotypes and, even in different patients with the same G306R homozygous mutation within a family, there are differences in the age of onset, clinical symptoms, and disease progression. This variable phenotype suggests that additional modifier genes or epigenetic factors may influence the final disease phenotype.

In the absence of treatment, Brown-Vialetto-Van Laere syndrome is a progressive and often fatal disease. Supplementation with riboflavin forms the foundation of disease treatment and is an important factor in determining the prognosis (*p* < 0.001). Through the review of the literature, we found that early riboflavin supplementation can significantly improve motor-sensory function, bulbar disturbances, respiratory disturbances, and hearing and vision functions, even for postnatal onset splice site variants.Karakaya et al. reported a case of a compound heterozygous patient with a splice site variant and a missense mutation, with onset after birth, in which the disease manifested as extensive muscle weakness, and respiratory dysfunction requiring ventilator-assisted breathing maintenance. After treatment with riboflavin, the respiratory disturbance resolved and the mechanical ventilator was eventually withdrawn (Karakaya et al., 2018). Early initiation of treatment may change the disease course and may also, for instance, prevent some patients from developing respiratory insufficiency. This might in some cases be a more important factor than the type of mutation. Delayed treatment is related to poor patient outcomes, especially in patients with early-onset disease (Bosch et al., 2012). At the start of riboflavin treatment, the present patient had severe sensorineural deafness which did not improve significantly, requiring cochlear implant assistance. These findings are consistent with those reported by Menezes (Menezes et al., 2016) and may be explained by degeneration of the retrocochlear and central auditory pathways (Sinnathuray et al., 2011). Previous autopsies of BVVL type 2 patients have identified neuropathological changes in the auditory pathway, including the brainstem, including neuronal degeneration in the cochlear nucleus and astrocytic gliosis in the inferior colliculus (Gallai et al., 1981; Francis et al., 1993). At present, the reported doses of riboflavin supplementation range from 7 mg/kg.d to 70 mg/kg.d but there is no conclusion about the dose that is sufficient to reverse or stop the continuing neuropathic damage. After treatment with low-dose riboflavin, the present patient’s symptoms improved, and the abnormal carnitine level returned to normal. However, evidence of demyelination was seen on the re-examination of the head MRI, indicating that the lesions were still insidious and slowly progressing. Therefore, there is an urgent need to identify a marker to reflect the pharmacodynamic relationship. Many studies have found that high-dose riboflavin supplementation has no obvious or fatal side effects (Haack et al., 2012; Foley et al., 2014; Horoz et al., 2016), so high-dose riboflavin supplementation is still recommended. For patients with gastrointestinal intolerance, the dose can be gradually increased from a small dose to reduce adverse reactions given that early supplementation can significantly improve prognosis. Therefore, if BVVL type 2 is suspected, there is no need to wait for genetic results, and early experimental supplementary intervention with riboflavin can be given.

### Potential bias

The analysis of the relationships between genotype and clinical phenotype presented here was mainly based on a literature review, and there is inevitably a certain bias in the extraction of clinical phenotypic and other data reported by different investigators. In addition, survival bias is also possible, as patients with severe phenotypes may be underrepresented due to neonatal or early infant mortality. Therefore, the results regarding the phenotype-genotype correlations of BVVL type 2 should be interpreted with caution.

## Conclusion

This study is the first to report a patient with BVVL type 2 caused by uniparental disomy of the *SLC52A2* gene, thus expanding the mutational spectrum. Furthermore, this study analyzed genotype-clinical phenotype correlations. This report helps to inform families about the range of symptoms associated with BVVL type 2.However We are afraid that reliable predictions based on the genotype can still not be made based on the results of the report. When a patient presents with symptoms of sensorimotor nerve damage accompanied by changes in hearing and vision, the possibility of BVVL type 2 should be considered. Early administration of riboflavin can delay disease progression and improve prognosis.

## Data Availability

The datasets for this article are not publicly available due to concerns regarding participant/patient anonymity. Requests to access the datasets should be directed to the corresponding author.
