# Supplementary material for: First report of paternal uniparental disomy of chromosome 8 with SLC52A2 mutation in Brown-vialetto-van laere syndrome type 2 and an analysis of genotype-phenotype correlations
Source: Front Genet. 2022 Sep 15;13:977914. doi: 10.3389/fgene.2022.977914 (PMC9520306; doi:10.3389/fgene.2022.977914)
Supplement: Supplementary file 1 [file Table1.DOCX]

**Supplementary Table. Clinical features of the present case and cases reported in the literature**

| **No.** | **Sex** | **Family** | **Onset**  **age（m）** | **Age at genetic diagnosis（m）** | **initial symptom** | **Visual loss** | **Optic atrophy** | **nystagmus** | **Hearing loss** | **bulbar palsy** | **weakness** | **respiratory insufficiency** | **ataxia** | **Riboflavin treatment** | **Age at riboflavin therapy(m)** | **Riboflavin dose** | **outcome** | **lost the ability to walk during follow-up** | **mutation 1** | **mutation 2** | **Genotype**  **by mutation**  **class** | **References** |
| --- | --- | --- | --- | --- | --- | --- | --- | --- | --- | --- | --- | --- | --- | --- | --- | --- | --- | --- | --- | --- | --- | --- |
| 1 | F | 1 | 12 | 21 | Lack of facial expression  hypotonia,weakness in upper limbs | + | NA | NA | + | + | + | + | NA | Yes | 21 | 50 mg/kg.d | Improvement | NA | c.1022_1023insC（p.Leu341Profs*103) | c.1328G>A (p.Cys443Tyr) | TM/EC | Shi et al., 2019 |
| 2 | M | 2 | 6 | NA | NA | NA | NA | NA | NA | NA | + | NA | NA | NA | NA | NA | Died at 12 mos | NA | c.696_703del  (p.Leu233Glyfs) | c.1250T>C (p.Leu417Pro) | IC/TM | Naess et al., 2021 |
| 3 | M | 3 | 12 | 72 | Nystagmus | NA | + | + | + | + | + | NA | NA | Yes | 53 | 30mg/kg.d | Alive | NA | c.1327T>C (p.Cys443Arg) | c.1327T>C (p.Cys443Arg) | EC/EC | Set et al., 2018 |
| 4 | F | 3 | 24 | 60 | Nystagmus | + | + | + | + | NA | + | NA | NA | Yes | 30 | 30 mg/kg.d | Alive | Unable to walk at 15 mos | c.1327T>C (p.Cys443Arg) | c.1327T>C (p.Cys443Arg) | EC/EC | Set et al., 2018 |
| 5 | F | 4 | 7 | 25 | Nystagmus | + | + | + | - | + | + | + | NA | NA | NA | NA | Died at 25 mos | NA | c.935T>C (p.Leu312Pro) | c.935T>C (p.Leu312Pro) | TM/TM | Manole et al., 2017 |
| 6 | F | 5 | Childhood | 324 | Hearing loss | + | + | NA | + | NA | + | + | NA | NA | NA | NA | Alive | Independent walking | c.383C>T (p.Ser128Leu) | c.1088C>T (p.Pro363Leu) | TM/EC | Manole et al., 2017 |
| 7 | M | 6 | 15 | 204 | Visual loss | + | + | NA | + | NA | + | + | NA | NA | NA | NA | Alive | Unable to walk | c.1016T>C (p.Leu339Pro) | c.935T>C (p.Leu312Pro) | TM/TM | Manole et al., 2017 |
| 8 | F | 7 | 36 | 648 | Hearing loss | + | + | NA | + | + | + | + | + | NA | NA | NA | Alive | Independent walking | c.231G>A (p.Glu77Lys) | c.865C>T (p.Ala288Val) | IC/TM | Manole et al., 2017 |
| 9 | M | 8 | 30 | 96 | Nystagmus | + | + | + | + | + | + | - | NA | NA | NA | NA | Alive | Unable to walk | c.1327T>C (p.Cys443Arg) | c.1327T>C (p.Cys443Arg) | EC/EC | Manole et al., 2017 |
| 10 | F | 9 | 18 | 48 | Visual loss | + | + | NA | + | + | + | - | NA | NA | NA | NA | Alive | NA | c.1327T>C (p.Cys443Arg) | c.1327T>C (p.Cys443Arg) | EC/EC | Manole et al., 2017 |
| 11 | F | 10 | 15 | 19 | Nystagmus | NA | NA | + | NA | NA | + | NA | + | Yes | 19 | 70 mg/kg.d | Improvement | Independent walking | c.1016T>C (p.Leu339Pro) | c.808C>T (p.Gln270∗) | TM/IC | Petrovski et al., 2015 |
| 12 | M | 11 | 30 | 96 | Abnormal gait | NA | NA | NA | + | + | + | + | + | No | - | - | Died at 132 mos | Unable to walk at 54 mos | c.916G>A (p.Gly306Arg) | c.916G>A (p.Gly306Arg) | EC/EC | Johnson et al., 2012 |
| 13 | M | 11 | 30 | 84 | Abnormal gait | NA | NA | + | + | + | + | NA | + | NA | NA | NA | Died at 88 mos | NA | c.916G>A (p.Gly306Arg) | c.916G>A (p.Gly306Arg) | EC/EC | Johnson et al., 2012 |
| 14 | F | 11 | 30 | NA | Abnormal gait | NA | NA | NA | + | NA | NA | NA | + | NA | NA | NA | Died at 48 mos | NA | c.916G>A (p.Gly306Arg) | c.916G>A (p.Gly306Arg) | EC/EC | Johnson et al., 2012 |
| 15 | M | 11 | 42 | 96 | Abnormal gait | NA | NA | NA | + | + | + | NA | + | NA | NA | NA | Alive | NA | c.916G>A (p.Gly306Arg) | c.916G>A (p.Gly306Arg) | EC/EC | Johnson et al., 2012 |
| 16 | M | 12 | 36 | 324 | Ataxia | + | NA | NA | + | NA | + | NA | + | No | - | - | Alive | Unable to walk at 300 mos | c.916G>A (p.Gly306Arg) | c.916G>A (p.Gly306Arg) | EC/EC | Guissart et al., 2016 |
| 17 | F | 12 | 36 | 72 | Ataxia | + | NA | NA | + | NA | NA | NA | + | No | - | - | Alive | NA | c.916G>A (p.Gly306Arg) | c.916G>A (p.Gly306Arg) | EC/EC | Guissart et al., 2016 |
| 18 | F | 13 | 120 | 156 | Ataxia | + | + | NA | + | NA | + | NA | + | Yes | 156 | 400mg/d | Alive | Independent walking | c.401C>T (p.Pro134Leu) | c.401C>T (p.Pro134Leu) | TM/TM | Guissart et al., 2016 |
| 19 | F | 13 | 24 | 108 | Ataxia | + | + | NA | + | + | + | NA | + | Yes | 108 | 400mg/d | Alive | Independent walking | c.401C>T (p.Pro134Leu) | c.401C>T (p.Pro134Leu) | TM/TM | Guissart et al., 2016 |
| 20 | M | 14 | NA | 95 | Ataxia | NA | NA | NA | + | NA | + | NA | + | Yes | 95 | 65mg/kg.d | Alive | NA | c.917G>A (p.Gly306Glu) | c.917G>A (p.Gly306Glu) | EC/EC | Cordeiro et al., 2018 |
| 21 | M | 15 | 18 | 60 | Mild language delay | + | + | + | + | - | + | - | NA | Yes | 60 | 50mg/kg.d | Improvement | Unable to walk  at 60 mos | c.505C>T (p.Arg169Cys) | c.505C>T (p.Arg169Cys) | TM/TM | Woodcock et al., 2018 |
| 22 | M | 15 | NA | 48 | Delayed development | NA | + | + | NA | NA | + | NA | NA | No | - | - | Died at 48 mos | Never acquired walking skills | c.505C>T (p.Arg169Cys) | c.505C>T (p.Arg169Cys) | TM/TM | Woodcock et al., 2018 |
| 23 | M | 16 | 25 | 25 | Macrocytic anemia | NA | NA | NA | NA | + | + | - | + | Yes | 25 | 70mg/kg.d | Improvement | Unable to walk  at 25 mos | c.1016T>C (p.Leu339Pro) | c.405_407delCTT (p.Phe135del) | TM/TM | Pillai et al., 2020 |
| 24 | F | 17 | 36 | 66 | Impaired hearing | + | + | + | + | NA | + | NA | + | Yes | 66 | 10mg/kg.d | Improvement | Unable to walk  at 60 mos | c.368T>C (p.Leu123Pro) | c.1016T>C (p.Leu339Pro) | TM/TM | Haack et al., 2012b |
| 25 | F | 18 | 36 | 240 | Ataxic | + | + | NA | + | NA | + | NA | + | No | - | - | Alive | Unable to walk  at 96 mos | c.916G>A (p.Gly306Arg) | c.916G>A (p.Gly306Arg) | EC/EC | Srour et al., 2014 |
| 26 | F | 18 | 42 | 204 | Ataxic | + | + | NA | + | + | + | NA | + | Yes | 204 | 10-15 mg/kg.d | Improvement | Unable to walk  at 165 mos | c.916G>A (p.Gly306Arg) | c.916G>A (p.Gly306Arg) | EC/EC | Srour et al., 2014 |
| 27 | M | 18 | 18 | 48 | Ataxic | - | - | NA | + | - | - | NA | + | Yes | 48 | 10-15 mg/kg.d | Improvement | Independent walking | c.916G>A (p.Gly306Arg) | c.916G>A (p.Gly306Arg) | EC/EC | Srour et al., 2014 |
| 28 | M | 18 | 30 | 228 | Ataxic | + | + | NA | + | + | + | + | + | No | - | - | Died at 264 mos | Unable to walk  at 216 mos | c.916G>A (p.Gly306Arg) | c.916G>A (p.Gly306Arg) | EC/EC | Srour et al., 2014 |
| 29 | M | 18 | 18 | 228 | Ataxic | - | - | NA | + | - | + | NA | + | Yes | 228 | 10-15 mg/kg.d | Improvement | Independent walking | c.916G>A (p.Gly306Arg) | c.916G>A (p.Gly306Arg) | EC/EC | Srour et al., 2014 |
| 30 | F | 19 | NA | 108 | Ataxia | NA | - | NA | - | NA | NA | - | + | Yes | 120 | NA | Alive | NA | c.916G>A (p.Gly306Arg) | c.916G>A (p.Gly306Arg) | EC/EC | Fogel et al., 2014 |
| 31 | M | 20 | 36 | 420 | Decline of hearing | + | + | NA | + | + | - | - | + | Yes | 420 | 1500mg/d | Improvement | NA | c.968T>C (p.Leu323Pro) | c.968T>C (p.Leu323Pro) | TM/TM | Gorcenco et al., 2019 |
| 32 | M | 21 | 24 | 39 | Dysphonia,notable exercise intolerance ,dyspnoea and cyanosis | + | NA | NA | + | + | + | + | NA | No | - | - | Died at 39 mos | Unable to walk  at 39 mos | c.155C>T (p.Ser52Phe） | c.1255G>A (p.Gly419Ser) | TM/TM | Ciccolella et al., 2013 |
| 33 | M | 22 | 54 | 96 | Ataxia | NA | NA | NA | + | NA | + | NA | + | Yes | 84 | 70 mg/kg.d | Improvement | Walking needs assistance at 84 mos | c.917G>A (p.Gly306Glu) | c.917G>A (p.Gly306Glu) | EC/EC | Nimmo et al., 2018 |
| 34 | M | 23 | 108 | 192 | Respiratory compromise | NA | NA | NA | + | + | + | + | NA | Yes | 192 | NA | Improvement | Independent walking | c.421C>A (p.Pro141Thr) | c.421C>A (p.Pro141Thr) | IC/IC | Udhayabanu et al., 2016 |
| 35 | F | 24 | 108 | 420 | Hearing loss | + | + | - | + | - | - | - | + | NA | NA | NA | Alive | Walking needs assistance | c.973T>G (p.Cys325Gly) | c.973T>G (p.Cys325Gly) | TM/TM | Babanejad et al., 2018 |
| 36 | F | 24 | 96 | 480 | Hearing loss | + | + | - | + | - | - | - | + | NA | NA | NA | Death | Walking needs assistance | c.973T>G (p.Cys325Gly) | c.973T>G (p.Cys325Gly) | TM/TM | Babanejad et al., 2018 |
| 37 | F | 24 | 108 | 324 | Hearing loss | + | + | - | + | - | - | - | + | No | - | - | Alive | Walking needs assistance | c.973T>G (p.Cys325Gly) | c.973T>G (p.Cys325Gly) | TM/TM | Babanejad et al., 2018 |
| 38 | F | 25 | 18 | 108 | Ataxia | - | - | - | - | - | - | NA | + | Yes | 108 | 10.5 mg/kg.d | Improvement | Walking needs assistance | c.916G>A (p.Gly306Arg) | c.916G>A (p.Gly306Arg) | EC/EC | Fan and Fogel, 2018 |
| 39 | F | 26 | 6 | 36 | Ataxia | NA | NA | NA | NA | NA | NA | NA | + | NA | NA | NA | NA | NA | c.505C>T (p.Arg169Cys) | c.505C>T (p.Arg169Cys) | TM/TM | Sun et al., 2019 |
| 40 | NA | 27 | birth | NA | Global muscle weakness | NA | NA | NA | NA | NA | + | + | NA | Yes | NA | NA | Improvement | NA | c.-110-1G>A (p.(?)(;)) | c.297G>C (p.Trp99Cys) | -/TM | Karakaya et al., 2018 |
| 41 | NA | 28 | NA | NA | Hearing loss | NA | NA | NA | + | NA | NA | NA | NA | NA | NA | NA | NA | NA | c.167C>T (p.Ala56Val) | c.593G>A (p.Trp198*) | TM/TM | van Beeck et al., 2019 |
| 42 | F | 29 | 4 | 72 | Nystagmus | + | + | + | + | NA | + | NA | + | Yes | NA | NA | NA | NA | c.1327T>C (p.Cys443Arg) | c.1327T>C (p.Cys443Arg) | EC/EC | Gahl et al., 2016 |
| 43 | M | 29 | 4 | 24 | Nystagmus | + | + | + | **+** | NA | + | NA | + | Yes | NA | NA | NA | NA | c.1327T>C (p.Cys443Arg) | c.1327T>C (p.Cys443Arg) | EC/EC | Gahl et al., 2016 |
| 44 | F | 30 | 18 | 120 | Ataxic gait | + | + | NA | + | NA | + | + | + | Yes | 127.2 | 50 mg/kg.d | Improvement | Walking needs assistance | c.916G>A (p.Gly306Arg) | c.916G>A (p.Gly306Arg) | EC/EC | Foley et al., 2014 |
| 45 | F | 31 | 12 | 72 | Ataxic gait | + | + | NA | + | + | + | + | + | Yes | 72 | 10 mg/kg.d | NA | Unable to walk  at 72 mos | c.92 G>C (p.Trp31Ser) | c.935T>C (p.Leu312Pro） | TM/TM | Foley et al., 2014 |
| 46 | F | 32 | 42 | 60 | Ataxic gait | + | + | NA | + | + | + | + | + | Yes | 64.8 | 7 mg/kg.d | Alive | Walking needs assistance | c.700C>T (p.Gln234*) | c.1258G>A (p.Ala420Thr） | IC/TM | Foley et al., 2014 |
| 47 | F | 33 | 18 | 60 | Right-sided ptosis | + | + | NA | + | NA | + | + | NA | Yes | 60 | 40 mg/kg.d | Improvement | Walking needs assistance | c.916G>A (p.Gly306Arg) | c.1016T>C (p.Leu339Pro） | EC/TM | Foley et al., 2014 |
| 48 | F | 34 | 36 | 144 | Weakness | + | NA | NA | + | + | + | + | NA | NA | NA | NA | NA | Walking needs assistance | c.916G>A (p.Gly306Arg) | c.1016T>C (p.Leu339Pro） | EC/TM | Foley et al., 2014 |
| 49 | F | 35 | 7 | 210 | Nystagmus | + | + | + | + | + | + | + | NA | Yes | 210 | 300 mg/d | Alive | Walking needs assistance | c.935T>C (p.Leu312Pro） | c.1016T>C (p.Leu339Pro） | TM/TM | Foley et al., 2014 |
| 50 | M | 36 | 24 | 258 | Hearing loss | + | + | NA | + | + | + | + | NA | Yes | 268.8 | 1500 mg/d | Improvement | Walking needs assistance | c.916G>A (p.Gly306Arg) | c.1258G>A (p.Ala420Thr） | EC/TM | Foley et al., 2014 |
| 51 | F | 37 | 96 | 120 | Ataxic gait | - | - | NA | + | NA | - | - | + | Yes | 120 | 23 mg/kg.d | Improvement | Independent walking | c.916G>A (p.Gly306Arg) | c.916G>A (p.Gly306Arg) | EC/EC | Foley et al., 2014 |
| 52 | F | 37 | 36 | 108 | Ataxic gait | + | + | NA | + | NA | + | - | + | Yes | 108 | 26 mg/kg.d | Alive | Independent walking | c.916G>A (p.Gly306Arg) | c.916G>A (p.Gly306Arg) | EC/EC | Foley et al., 2014 |
| 53 | F | 38 | 24 | 42 | Ataxic gait and weakness | NA | NA | NA | NA | NA | + | + | + | NA | NA | NA | NA | Walking needs assistance | c.916G>A (p.Gly306Arg) | c.1016T>C (p.Leu339Pro） | EC/TM | Foley et al., 2014 |
| 54 | F | 38 | 60 | 180 | Hearing loss | + | + | NA | + | NA | + | + | NA | Yes | 180 | 21 mg/kg.d | Alive | Independent walking | c.916G>A (p.Gly306Arg) | c.1016T>C (p.Leu339Pro） | EC/TM | Foley et al., 2014 |
| 55 | M | 39 | 36 | 192 | Ataxic gait and hearing loss | + | + | NA | + | + | + | + | + | Yes | 192 | 1000 mg/d | Improvement | Walking needs assistance | c.916G>A (p.Gly306Arg) | c.916G>A (p.Gly306Arg) | EC/EC | Foley et al., 2014 |
| 56 | M | 39 | 36 | 192 | Ataxic gait and hearing loss | + | + | NA | + | + | + | + | + | Yes | 192 | 1000 mg/d | Alive | Walking needs assistance | c.916G>A (p.Gly306Arg) | c.916G>A (p.Gly306Arg) | EC/EC | Foley et al., 2014 |
| 57 | M | 39 | 60 | 252 | Ataxic gait | + | + | NA | + | NA | + | - | + | Yes | 252 | 1000 mg/d | Alive | Independent walking | c.916G>A (p.Gly306Arg) | c.916G>A (p.Gly306Arg) | EC/EC | Foley et al., 2014 |
| 58 | F | 40 | 24 | 624 | Respiratory failure | + | + | NA | + | NA | + | + | NA | Yes | 624 | 400 mg/d | Alive | Independent walking | c.851C>A (p.Ala284Asp） | c.916G>A (p.Gly306Arg) | TM/EC | Foley et al., 2014 |
| 59 | M | 40 | 48 | 528 | Vision loss | + | + | NA | + | NA | + | - | NA | Yes | 528 | 800 mg/d | Alive | Walking needs assistance | c.851C>A (p.Ala284Asp） | c.916G>A (p.Gly306Arg) | TM/EC | Foley et al., 2014 |
| 60 | M | 41 | 15.6 | 22.8 | Nystagmus | + | + | + | + | + | + | + | NA | Yes | 21.6 | 60 mg/kg.d | Improvement | Only sitting at 23 mos | c.914A>G (p.Tyr305Cys） | c.916G>A (p.Gly306Arg) | EC/EC | Foley et al., 2014 |
| 61 | M | 42 | 36 | 72 | Hearing loss | NA | NA | NA | + | NA | + | - | NA | Yes | 78 | 10 mg/kg.d | Improvement | Independent walking | c.916G>A (p.Gly306Arg) | c.916G>A (p.Gly306Arg) | EC/EC | Foley et al., 2014 |
| 62 | M | 43 | 36 | 48 | Vision loss | + | + | + | + | + | + | - | + | Yes | 48 | 7 mg/kg.d | Improvement | Independent walking | c.1255G>A (p.Gly419Ser) | c.1255G>A (p.Gly419Ser) | TM/TM | Present case |

mo=month, NA= not available,TM=transmembrane region, EC=extracellular region,IC=intracellular region
